# Evaluation of Acetaminophen Release from Biodegradable Poly (Vinyl Alcohol) (PVA) and Nanocellulose Films Using a Multiphase Release Mechanism

**DOI:** 10.3390/nano10020301

**Published:** 2020-02-10

**Authors:** Kelsey L. O’Donnell, Gloria S. Oporto-Velásquez, Noelle Comolli

**Affiliations:** 1 Department of Chemical and Biological Engineering, Villanova University, 216 White Hall, Villanova, PA 19085, USA; noelle.comolli@villanova.edu; 2 Department of Forestry and Natural Resources, West Virginia University, 329C Percival Hall, Morgantown, WV 26506, USA; gloria.oporto@mail.wvu.edu

**Keywords:** nanocellulose, drug delivery, acetaminophen, TEMPO nanocellulose, poly (vinyl alcohol)

## Abstract

Biodegradable polymers hold great therapeutic value, especially through the addition of additives for controlled drug release. Nanocellulose has shown promise in drug delivery, yet usually requires chemical crosslinking with harsh acids and solvents. Nanocellulose fibrils (NFCs) and 2,2,6,6-tetramethylpiperidine-N-oxyl (TEMPO)-mediated oxidized nanocellulose fibrils (TNFCs) with poly (vinyl alcohol) (PVA) could be aqueously formulated to control the release of model drug acetaminophen over 144 h. The release was evaluated with a multiphase release mechanism to determine which mechanism(s) contribute to the overall release and to what degree. Doing so indicated that the TNFCs in PVA control the release of acetaminophen more than NFCs in PVA. Modeling showed that this release was mostly due to burst release—drug coming off the immediate surface, rather than diffusing out of the matrix.

## 1. Introduction

Controlled drug delivery is of high therapeutic value because it can extend the release time of a single dosage. Popular drug delivery platforms include nanoparticles, tablets, films, and transdermal patches. In the case of films and patches, biodegradable polymers such as poly (lactic-co-glycolic acid) (PLGA), poly (vinyl alcohol) (PVA), polylactide (PLA), polyglycolide (PGA), and poly (ε-caprolactone) (PCL) have been used as biocompatible matrices [1]. Copolymers, additives, and plasticizers are often formulated into these matrices to increase characteristics such as solubility, stability, and mechanical properties [2]. 

Cellulose, the most abundant renewable biopolymer, has been incorporated into a wide range of consumer products in various forms. Cellulose’s unique characteristics include, but are not limited to, surface chemical reactivity, biocompatibility, low toxicity, and mechanical properties [3,4,5,6,7,8,9,10]. Nanocellulose, as an example of cellulose forms, is produced after raw cellulosic material has undergone chemical and/or mechanical processes. Nanocellulose is typically classified into three major categories: 1) cellulose nanocrystals (CNCs), 2) nanofibrillated cellulose (NFC), and 3) bacterial cellulose (BC) [3,4,5,6,8]. NFC, the raw material used in this research, is typically produced from kraft bleached pulps using a mechanical and/or enzymatic treatment. The final nanofibrillated material has distinctive dimensions that range between 3 and 50 nm in diameter and several micrometers in length [6,8,11,12,13,14]. Carboxyl groups can be introduced during the NFC’s processing into the cellulosic backbone to improve delamination of the nanofibrils through 2,2,6,6-tetramethylpiperidine-N-oxyl (TEMPO)-mediated oxidation [11,12,13,15]. The TEMPO radical is a free radical that selectively oxidizes the primary alcohol group of polysaccharides in the presence of NaBr/NaClO [11,13,15]. The TEMPO/NaBr/NaClO treatment is applied to never-dried cellulose fibers prior to nanoscale processing to produce TEMPO-oxidized NFC (TNFC). This produces a more translucent hydrogel material from pulp-like NFC suspensions.

Nanocellulose as a drug delivery platform has been used in the form of hydrogels, where NFCs and CNCs can be formulated as suspensions, particles, gels, or composite biopolymer delivery systems [4,6,14,16,17]. Drug entrapment using NFCs or CNCs commonly involves crosslinking and/or surface modifications [4,5,6,16,17,18]. Drug-loaded matrices include cellulose as suitable tablets for oral administration. In many of these formulations, nanocellulose is able to control the rate of drug release and deliver the appropriate drug concentration over time [19]. 

Mathematical models have long been used to predict the release behavior of drugs in order to aid optimal formulations and the design of new systems [1,20,21,22,23,24,25]. Many existing models focus on diffusional release as described by Fick’s law of diffusion [20,22,23]. Although applicable to drug transport through thick slabs, cylinders, and spheres, this approach accounts for one simple mechanism of release that is highly dependent on the matrix structure. As precise experimental data and observations are applied to diffusional models, the need for more sophisticated mathematical models becomes apparent. In addition to diffusional release, several other controlled release mechanisms include chemically controlled, osmotically controlled, and swelling and/or dissolution controlled [20,22]. More specific types of controlled release mechanisms incorporate polymer morphology and the internal structure through which the drug moves to reach the surrounding environment as well as properties specific to the chosen material (i.e., polarity, crystallinity, viscosity, molecular weight, additives) [2,20,23,25]. 

Chemically controlled release accounts for erodible and pendant chain systems, in which the drug release rate is dependent on polymer degradation and hydrolytic or enzymatic degradation, respectively. Erodible systems can dominate over diffusional through surface or bulk erosion. This is when polymer degradation occurs due to water moving quickly into a matrix of less-hydrophobic polymers (bulk erosion) or if the water is excluded from the bulk of the hydrophobic polymer matrix (surface erosion) [20,22]. Osmotically controlled release occurs when local osmotic pressure becomes sufficiently high to cause the system to rupture. Polymers are typically homogenously loaded with highly soluble drugs to be released from encapsulated spheres in a single pulse [20,22]. 

In swelling-controlled release, the polymer is placed into water or buffer and the solvent diffuses into the polymer causing volume expansion to release water-soluble drug from the matrix [20,22]. However, there are two interfaces within this system: the polymer interface contacting water moving outward, and the swelling interface moving inward as the matrix swells. During this swelling interface, polymer chains undergo relaxation and affect drug diffusion (Fickian or non-Fickian) through the polymer [20]. Acknowledging the effect of this on overall diffusion constructs a more realistic model of drug release. During swelling and relaxation, polymer chains uncrosslink, become disentangled, and dissolve in the surrounding solvent [20,22,24,25,26]. This is especially the case for biodegradable materials such as those that have been mentioned above. 

With biodegradable polymers incorporating cellulose, a majority of the literature reports biphasic release profiles, specifically for water-soluble drugs and small molecules [4]. The first phase is a quick initial burst in the first few hours followed by a prolonged diffusional release for up to 72 h [4,23,25,27,28,29]. However, incorporating the dissolution/relaxation mechanism would allow mathematical models to more accurately reflect the true driving forces that control the flux of drug through and out of the delivery platform [1]. 

In this research, an independent evaluation of NFC and TNFC as drug delivery platforms for acetaminophen (a model drug) was performed. The main hypothesis of this work was that carboxylic groups present in TNFC will improve the availability of the drug to be released. Biodegradable composites films using polyvinyl alcohol in combination with NFC and TNFC and acetaminophen, as purely aqueous formulations, were compared based on their prolonged release of acetaminophen. No chemical linkers or surface modifiers were used to prepare the suspensions and corresponding films. Collected experimental data were then evaluated with a tri-phasic release model based upon Lao, Venkatraman, and Peppas 2008 to determine the potential mechanisms and the degree to which these mechanisms contributed based on the formulation. 

## 2. Materials and Methods 

### 2.1. Materials

Nanofibrillated cellulose suspension (NFC, 3.0 wt%) supplied by the University of Maine, Orono, Maine, USA; TEMPO-oxidized nanofibrillated cellulose (TNFC, 2.0 wt%) supplied by the Forest Products Laboratory, Madison, WI, USA; Poly (vinyl alcohol) (PVA, 88 mol% hydrolyzed MW: ~78,000) from PolySciences, Inc, Warrington, PA, USA.; Acetaminophen (meets USP testing specifications, 98.0%–102.0% powder) from Sigma-Aldrich, St. Louis, MO, USA. 

### 2.2. Film Formulations

**Figure 1:**
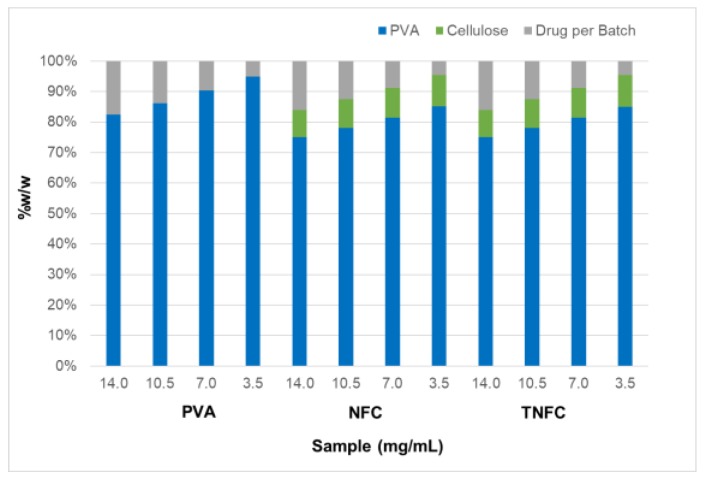
Formulations of poly (vinyl alcohol) (PVA), nanofibrillated cellulose (NFC)/PVA, and 2,2,6,6-Tetramethylpiperidine-N-oxyl (TEMPO)- oxidized NFC (TNFC)/PVA samples based on *w*/*w*%.

Four stock solutions of acetaminophen were made in deionized H_2_O based on acetaminophen’s maximum solubility—100% at 14.0 g/L, 75% at 10.5 g/L, 50% at 7.0 g/L, and 25% at 3.5 g/L (Table 1). To form the films, NFC and TNFC were separately added to acetaminophen solutions. PVA was then slowly added to the cellulose-drug mixtures in a 1:4 ratio (PVA:cellulose) with the specified type of cellulose (Figure 1) [28]. The mixtures were stirred for 20 min unheated, then heated to 90 °C and stirred for an additional 20 min. Heat was turned off for a final 10 min of mixing. Films were produced through solvent casting (7.5 cm × 2.5 cm × 0.2 cm) and oven-dried overnight at 40 °C. 

### 2.3. Drug Release Studies

Release studies were conducted using the SR-8 Plus Dissolution System from Hanson Research. Conditions were set at 37 °C and an agitation speed of 25 rpm. Samples with 2.5 cm × 2.5 cm dimensions were cut from film strips (7.5 cm × 2.5 cm × 0.2 cm) and placed in 1 L of phosphate-buffered saline (PBS) in individual dissolution cells. Samples (1 mL) were taken at 0 and 30 min, then every hour for the first 24 h. Samples were then taken every 12 h until day 6. The total sample (1 mL) was replaced with fresh filtered PBS buffer after every sampling (Millex – GN 0.20 µm Nylon Membrane Filter Unit). Samples were quantified using high-performance liquid chromatography (Shimadzu, reversed-phase C18) at 254 nm.

### 2.4. Drug Release Quantification

High-performance liquid chromatography (HPLC) (Shimadzu UFLC, Japan) was used with a premier C18 reverse-phase column (Shimadzu 50 mm × 4.6 mm, 3 µm particle diameter) to quantify acetaminophen in phosphate-buffered saline (PBS) from release samples. Injection volume was 20 µL with an absorption length of 254 nm and flow rate of 2.0 mL/min. 

### 2.5. Drug Release Modeling

Mathworks MATLAB R2018a was used to model drug release from the prepared biodegradable films. As described by Lao et al. [1], this mechanistic and modeling approach follows three steps: 1) solvent (water) penetration into the matrix causing a burst release; 2) a degradation-dependent “relaxation of the network” that creates more free volume for drug dissolution; and 3) drug removal to the surrounding medium, usually by diffusion [1]. Together, these three mechanisms combine to a triphasic release comprised of burst release, relaxation-induced drug dissolution release, and diffusion-controlled release. Each step is incorporated into Equation (1). Each term includes a fraction of drug released (*Φ*) and constant value (*k*), designated by the subscripts *b* for burst release, *r* for relaxation-induced dissolution release, and *d* for diffusion-controlled release.

(1) $$\frac{M_{t}}{M_{\infty }}=\Phi_{b}\left\{1-exp\left(-k_{b}t\right)\right\}+\Phi_{r}\left\{exp\left(k_{r}\left(t-t_{b}\right)\right)\right\}\;\;+\Phi_{d}\left\{1-\;\overset{\infty }{\underset{n=0}{\sum }}\frac{8}{{\left(2n+1\right)}^{2}\pi^{2}}exp\left(\frac{-D{\left(2n+1\right)}^{2}\pi^{2}\left(t-t_{r}\right)}{4l^{2}}\right)\right\}$$

Experimental data were initially fit to the burst term (Equation (2)), the first term in Equation (1). The imported data were calculated as the average values of each time point from six identical studies (*n* = 6). The fraction of burst release (*Φ_b_*), constant of burst release (*k_b_*), and R^2^ values were calculated based on this application.

(2) $$\frac{M_{t}}{M_{\infty }}=\Phi_{b}\left\{1-exp\left(-k_{b}t\right)\right\}$$

## 3. Results and Discussion

**Figure 2:**
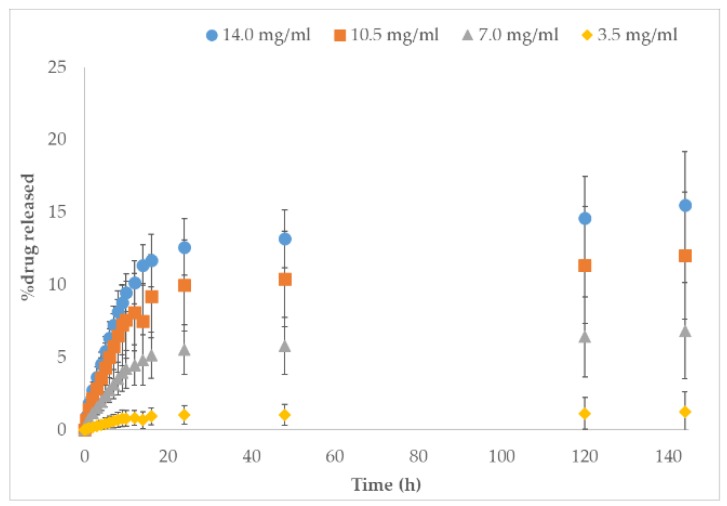
Comparative release profile based of NFC/PVA films over 144 h (*n* = 6) in varying acetaminophen concentrations: 14.0 mg/mL, 10.5 mg/mL, 7.0 mg/mL, and 3.5 mg/mL.

Repeated release studies were conducted for all formulations (*n* = 6). Average values and standard deviations were calculated and plotted under the estimation of 75% drug entrapment in the matrix. Figure 2 illustrates the varied acetaminophen concentration in NFC/PVA films. As expected, the highest concentration had the highest percent of acetaminophen released (15.5% ± 3.7%). Lower concentrations released acetaminophen in descending order: 15.5% ± 3.7%, 12.0% ± 4.4%, and 1.3% ± 1.3%. This demonstrates that when there is more drug in the formulation, more drug is released over time. The same trends were also seen in the control (PVA) and TNFC/PVA formulations (Table 2). However, the lack of release near 100% of drug could indicate that the drug was stuck in the polymeric matrix. This could be due to the innate fibril structure of alternating amorphous and crystalline regions entangling and trapping drug. 

**Figure 3:**
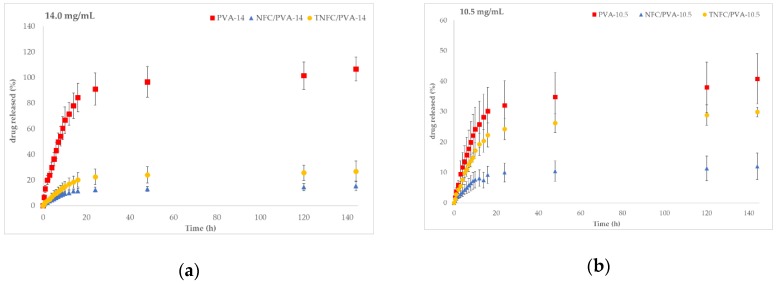

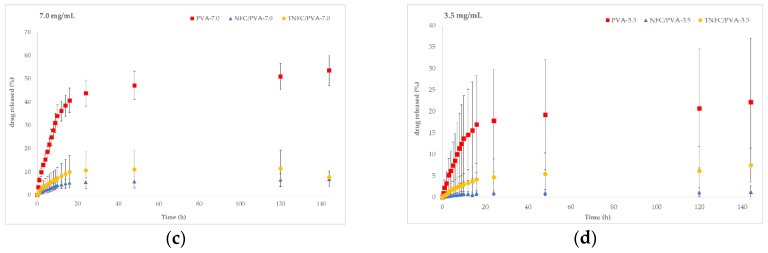
Comparative release profiles based on formulation (PVA (square), NFC/PVA (circle), TNFC/PVA (triangle)) for different initial acetaminophen concentrations: (**a**) 14.0 mg/mL, (**b**) 10.5 mg/mL, (**c**) 7.0 mg/mL, (**d**) 3.5 mg/mL.

Figure 3 compares the three formulations (PVA, NFC/PVA, and TNFC/PVA) based on drug concentration. Each plot shows the same release trend based on formulation: control (PVA) had the highest release, followed by TNFC/PVA, then NFC/PVA (above 100% release likely due to experimental error). Adding either type of cellulose to PVA films decreased drug release due to added resistance to the matrix. However, adding TNFC showed a greater release than adding NFC. The addition of TNFC increased the release of acetaminophen by 11.4% with 14 mg/mL, 17.8% with 10.5 mg/mL, 0.8% with 7.0 mg/mL, and 6.2% with 3.5 mg/mL compared to the addition of NFC (Table 2). The increased release with TNFC versus NFC was likely due to their innate structures and interactions with acetaminophen. TNFC is the product of TEMPO-oxidation of NFC, a common modification to cellulose during its processing to nanofibrils [12,15,29,30,31,32]. TEMPO is selective for primary alcohols (OH) and converts them to carboxylic acids (COOH) to change the surface reactivity. In acetaminophen, the hydrogen on the amine group has a pKa of 7.2. In the release environment of pH 7.4, this hydrogen leads to a partial positive charge (δ^+^) on the nitrogen and a partial negative charge (δ^−^) on the neighboring oxygen. The distribution of partial charges surrounded by the hydroxyl groups in PVA and the combination of innate structures and interactions with additional hydroxyls and carboxylic groups support the idea of better physical crosslinking in the matrix between TNFC and acetaminophen, leading to higher loading and higher release. Chemical crosslinking was disproved with FTIR spectra.

**Figure 4:**
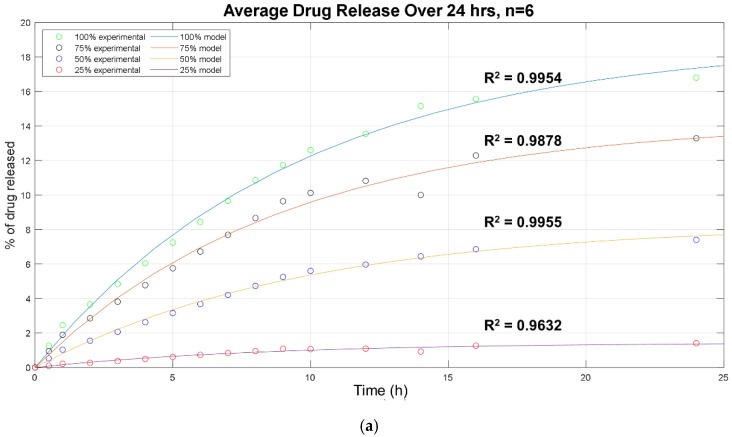

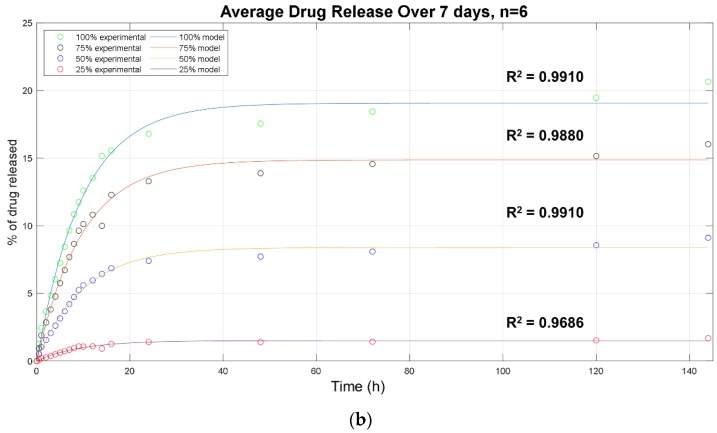
Acetaminophen release data from NFC/PVA film formulation (*n* = 6). Experimental measurements of drug release using HPLC are indicated by colored circles. Modeled releases using Equation (2) are represented by solid lines and labeled with their respective R^2^ values. Plots were produced in MATLAB. (**a**) 24-h release (**b**) full 144-h (6 days) release.

To further evaluate and understand these differences in release between the formulations, experimental data sets were fit to the first term of Equation (1) (simplified to Equation (2)). The imported data were the average values of each time point from identical studies of control, NFC/PVA, and TNFC/PVA, fitting for just the burst duration of the mechanism. Fits produced an average R^2^ value of 0.98 ± 0.01. These significant values indicate the occurrence of a burst release during this 24-h period (Figure 4a). From this, the fraction of burst release (*Φ_b_*) and burst constant (*k_b_*) were determined as well (Table 3). 

As presented in Figure 4 and Table 3, the fraction of burst release increased with increasing drug concentration in the initial formulations. This accounts for drug attached to the surface of the film and release, rather than being entrapped deeper in the polymer matrix. Therefore, even if more drug is released, it is not necessarily embedded into the polymer matrix and could instead simply be attached to the surface. The burst constant (*k_b_*) value remained constant between formulations at 0.10 ± 0.01. The increased burst is not unexpected, and depending on the application may be of clinical value. However, when fitting the entire 6-day sampling period to Equation (2), the modeled lines shift slightly, indicating the possible presence of another release mechanism (Figure 4b). The same trends were observed when fitting control (PVA) and TNFC/PVA to the same time periods.

**Figure 5:**
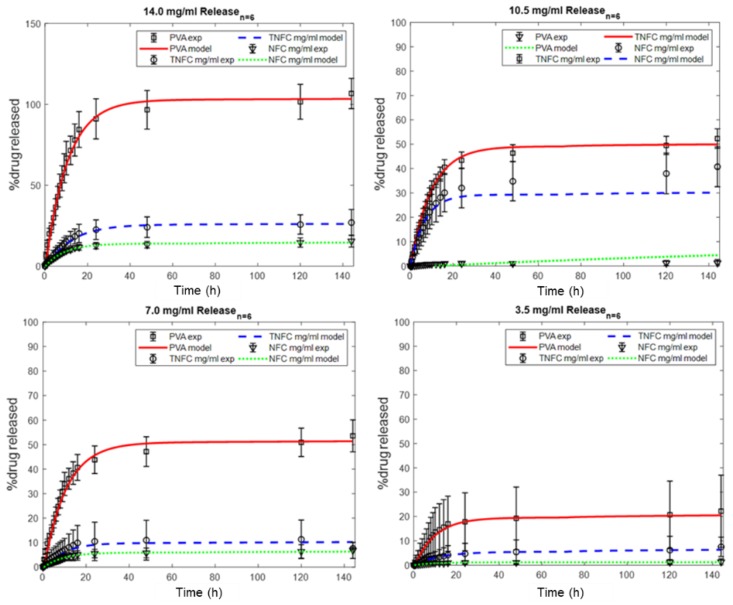
Fitting of triphase release mechanism (Equation (1)) to experimental drug release data based on drug concentration: 14.0 mg/mL (top left), 10.5 mg/mL (top right), 7.0 mg/mL (bottom left), and 3.5 mg/mL (bottom right).

Physically, this indicates that drug was being released from the outer layer of the film as the solution penetrated the matrix. Since, at most, only about 14% (NFC) and about 28% (TNFC) of the drug was released in this way (as seen in Figure 2a), the remaining amount must have been released through a secondary and possibly third mechanism, as proposed. This would include the drug moving through the matrix via diffusion and/or through a relaxation-induced dissolution mechanism. Another possibility is that the drug was entangled in the matrix, attributed to the alternating amorphous and crystalline regions characteristic of NFC. This entanglement could affect the drug’s rate of diffusion through the film, the penetration of water into the film, the amount of free volume for the drug to maneuver, and the dissolution rate of the film. When fitting the entirety of Equation (1) to the experimental release data (Figure 5), the remaining fractions (*Φ_r_*, *Φ_d_*) and constants (*k_r_*, *D*) can be determined (Table 4).

The quantification of three fractional releases indicates burst release, relaxation-induced dissolution release, and diffusion-controlled release were present in all three formulations. In all the formulations, burst release dominated the mechanism, which is characteristic of acetaminophen. The diffusion-controlled release showed the second-highest fraction, followed by relaxation-induced dissolution. Although the dissolution fractions were fairly small compared to the other two fractions, they were still present and indicative of dissolution being a contributing mechanism to drug release.

## 4. Conclusions

Polyvinyl alcohol film formulations were varied in terms of both material (NFC vs. TNFC) and drug concentration without using any chemical linkers. Physical crosslinking between nanocellulose and PVA proved to create a functional matrix for the release of acetaminophen. Release profiles for each followed the same trend: the more drug incorporated into the formulation, the greater the percent drug released. The control PVA formulation had the highest release due to the lack of cellulose and resistance in the matrix. TNFC had a higher release when added to PVA compared to NFC. NFC had about 50% less drug released compared to TNFC films for most of the concentrations. This shows that TNFC/PVA created less resistance in the matrix and a more controlled release of acetaminophen when evaluated based on percent drug released. 

A triphasic mathematical model was applied to determine the presence and degree of mechanisms occurring within these films. Control PVA and TNFC/PVA films demonstrated mainly diffusion-controlled and burst release, with extremely small fractions of relaxation-induced dissolution and prolonged-diffusional release. NFC/PVA films also showed mainly diffusion-controlled burst release, but a more significant presence of the burst and relaxation-induced dissolution mechanisms. The presence of each mechanism supports the need to incorporate additional phases into the mathematical model and expand on the traditionally accepted diffusional model of solely burst and diffusion based release for nanocellulose composites.

Interpreting both the drug release percentages and mechanisms, it is proposed the drug is entangled in the matrix, attributed to the alternating amorphous and crystalline regions characteristic of NFC. This entanglement could be greater with NFC than TNFC and could affect the drug’s rate of diffusion through the film, the penetration of water into the film, the amount of free volume for the drug to maneuver, and the dissolution rate of the film. This creates a basis for incorporating other molecules in the same formulations, evaluating their release mechanisms, and quantifying the amount released over time. 

## Figures and Tables

**Table 1:** Calculation of stock acetaminophen solution concentrations used in film formulations.

Drug (mg)	dI-H_2_O (mL)	Stock (mg/mL)
4203.0	300.0	14.0
3154.0	300.0	10.5
2106.0	300.0	7.0
1050.0	300.0	3.5

**Table 2:** Ending drug release percentage from PVA, NFC, and TNFC films at each drug concentration after 144 h.

Sample	14.0 mg/mL	10.5 mg/mL	7.0 mg/mL	3.5 mg/mL
PVA	106.7% ± 9.4%	40.7% ± 8.2%	53.5% ± 6.5%	22.2% ± 14.8%
NFC	15.5% ± 3.7%	12.0% ± 4.4%	6.8% ± 3.3%	1.3% ± 1.3%
TNFC	26.9% ± 8.0%	29.8% ± 1.6%	7.6% ± 0.4%	7.5% ± 3.9%

**Table 3:** Values related to the burst release of each formulation, determined by fitting Equation (2) to the experimental data in MATLAB.

Sample	*Φ_b_*	*k_b_*	R^2^
PVA-14.0	105.5	0.09	0.99
PVA-10.5	49.4	0.10	1.00
PVA-7.0	53.6	0.10	0.99
PVA-3.5	20.7	0.10	0.99
NFC/PVA-14.0	14.2	0.10	1.00
NFC/PVA-10.5	10.7	0.11	0.99
NFC/PVA-7.0	6.2	0.10	1.00
NFC/PVA-3.5	1.2	0.11	0.99
TNFC/PVA-14.0	27.5	0.08	1.00
TNFC/PVA-10.5	29.3	0.08	0.99
TNFC/PVA-7.0	12.2	0.09	0.99
TNFC/PVA-3.5	5.5	0.08	0.99

**Table 4:** Values estimated in MATLAB from fitting the multiphase release equation (Equation (1)) to experimental data. MATLAB inputs were average values from each time point.

Sample	*k_b_* (day^−1^)	*Φ_b_* (%)	*k_r_* (day^−1^)	*Φ_r_* (%)	*D* (10^−12^cm^2^/s)	*Φ_d_* (%)	Total (%)
PVA-14.0	3.7	105.5	0.1	0.1	0.63	0.0	141.2
PVA-10.5	3.9	49.4	0.0	0.0	62.9	0.1	50.8
PVA-7.0	3.5	53.6	0.0	0.0	62.9	0.1	65.8
PVA-3.5	2.2	20.7	0.0	0.1	0.63	0.0	12.2
NFC/PVA-14.0	2.6	14.2	0.0	0.0	62.9	0.1	14.3
NFC/PVA-10.5	2.7	10.7	0.0	0.0	62.9	0.1	10.8
NFC/PVA-7.0	2.6	6.2	0.5	0.0	33.3	0.1	6.3
NFC/PVA-3.5	2.9	1.2	0.5	0.0	1.4	0.1	1.2
TNFC/PVA-14.0	1.9	27.5	0.7	0.0	0.7	0.0	27.5
TNFC/PVA-10.5	2.1	29.3	0.6	0.0	62.8	0.1	29.4
TNFC/PVA-7.0	2.4	12.2	0.9	0.0	0.6	0.0	12.2
TNFC/PVA-3.5	1.9	5.5	0.0	0.0	62.9	0.1	5.6
